# To Be or Not To Be…Toxic—Is RNA Association With TDP-43 Complexes Deleterious or Protective in Neurodegeneration?

**DOI:** 10.3389/fmolb.2019.00154

**Published:** 2020-01-10

**Authors:** Suvithanandhini Loganathan, Erik M. Lehmkuhl, Randall J. Eck, Daniela C. Zarnescu

**Affiliations:** ^1^Department of Molecular and Cellular Biology, University of Arizona, Tucson, AZ, United States; ^2^Department of Neuroscience, University of Arizona, Tucson, AZ, United States

**Keywords:** TDP-43, transactive response DNA-binding protein 43, neurodegenaration, ALS (amyotrophic lateral sclerosis), RNA, stress granules (SG)

## Abstract

TAR DNA binding protein (TDP-43) is a nucleic acid binding protein associated with insoluble cytoplasmic aggregates in several neurodegenerative disorders, including 97% of the ALS cases. In healthy individuals, TDP-43 is primarily localized to the nucleus; it can shuttle between the nucleus and the cytoplasm, and is involved in several aspects of RNA processing including transcription, splicing, RNA stability, transport, localization, stress granule (SG) formation, and translation. Upon stress, TDP-43 aggregates in the cytoplasm and associates with several types of RNA and protein assemblies, resulting in nuclear depletion of TDP-43. Under conditions of prolonged stress, cytoplasmic TDP-43 undergoes liquid-liquid phase separation (LLPS) and becomes less mobile. Evidence exists to support a scenario in which insoluble TDP-43 complexes sequester RNA and/or proteins causing disturbances in both ribostasis and proteostasis, which in turn contribute to neurodegeneration. However, the relationship between RNA binding and TDP-43 toxicity remains unclear. Recent studies provide conflicting views on the role of RNA in TDP-43 toxicity, with some finding RNA as a toxic factor whereby RNA binding contributes to TDP-43 toxicity, while others find RNA to be a protective factor that inhibits TDP-43 aggregation. Here we review and discuss these recent reports, which ultimately highlight the importance of understanding the heterogeneity of TDP-43 assemblies and collectively point to solubilizing TDP-43 as a potential therapeutic strategy.

## Introduction

Although deficiencies in several dozen genes have been associated with ALS (Peters et al., 2015), a great proportion of patients have no known ALS associated mutation and disease etiology remains poorly understood. Multiple phenotypes including metabolic dysfunction (Dupuis et al., 2011; Joardar et al., 2017), ER stress, proteasome defects (Walker and Atkin, 2011; Karademir et al., 2015), and altered RNA processing (Ling et al., 2013; Taylor and Brown, 2016; Yasuda and Mili, 2016) have been implicated in disease pathomechanisms. However, the causal order and interdependent relationships between these phenotypes remains unclear, in part because the early events are missed by the time a diagnosis is made. A key unifying feature of disease is the DNA/RNA binding protein TDP-43, which is found in cytoplasmic aggregates in 97% of ALS and 45% of FTD cases regardless of etiology. Superoxide dismutase 1 (SOD1, 2%) and Fused in Sarcoma (FUS, <1%) pathologies are involved in the rest of ALS cases while TAU (45%) and FUS (9%) pathologies makes up the remaining FTD pathologies (Ling et al., 2013). In addition, TDP-43 aggregates have been reported in up to 50% of Alzheimer's disease cases (Chang et al., 2016), chronic traumatic encephalopathy (Ling H. et al., 2015), and observed at low frequency in Parkinson's (Markopoulou et al., 2008) and Huntington's Diseases (Schwab et al., 2008). Furthermore, 2–4% of ALS patients harbor mutations in TDP-43, which together with the presence of wild-type TDP-43 in pathological aggregates highlight its role as a common denominator for the majority of ALS cases and a significant fraction of related neurodegenerative disorders. Since its discovery in 2006 as a major component of pathological aggregates, we have learned a great deal about the involvement of TDP-43 in several steps of RNA processing including transcription, splicing, RNA stability, transport, localization, and translation, with several excellent reviews being recently written on the role of TDP-43 in these processes (Bowden and Dormann, 2016; Coyne et al., 2017b; Butti and Patten, 2018; Lehmkuhl and Zarnescu, 2018; Afroz et al., 2019; Birsa et al., 2019; Hergesheimer et al., 2019); however, its precise involvement in disease remains to be elucidated. Despite the presence of two RNA binding domains (RNA Recognition Motifs, RRM1 and 2) within its structure (Buratti and Baralle, 2001) and numerous reports of binding to specific RNA sequences and mRNA targets (Polymenidou et al., 2011; Sephton et al., 2011; Tollervey et al., 2011; Arnold et al., 2013), the significance of RNA association with TDP-43 and its contribution to disease pathology remains a topic of debate. Here we review recent molecular, imaging and pharmacological data on the relationship between RNA and TDP-43 complexes and discuss the possible contributions of RNA-TDP-43 interactions to disease pathology.

## TDP-43 Structure, Localization, and Function

### Structural Features of TDP-43

TDP-43 is a highly conserved 414-amino-acid protein, encoded by the TARDBP gene (Ayala et al., 2005), originally identified as a transcriptional repressor that binds the TAR-DNA sequence of the human immunodeficiency virus 1 (HIV-1) (Ou et al., 1995). It was subsequently shown that TDP-43 binds to RNA containing UG repeats and promotes skipping of exon 9 in the cystic fibrosis transmembrane conductance regulator (CFTR) pre-mRNA (Buratti et al., 2001, 2004). TDP-43 contains several domains including an N terminus harboring a nuclear localization signal (NLS), two RNA recognition motifs (RRM1 and RRM2), a nuclear export signal (NES), and a C- terminus comprising a glutamine/asparagine-rich (Q/N) domain and a glycine rich region (Ayala et al., 2008).

The N terminal domain has been shown to play a role in the dimerization of TDP-43 (Chang et al., 2012; Afroz et al., 2017). Both RRM domains of TDP-43 bind nucleic acids with high specificity toward UG rich RNA and TG rich DNA sequences (Buratti and Baralle, 2001; Kuo et al., 2009, 2014; Lukavsky et al., 2013). Mutation analyses show that RRM1 and RRM2 are both required for RNA/DNA binding comprising UG/TG stretches, with RRM1 playing a predominant role while RRM2 has more of a supporting role (Buratti and Baralle, 2001; Kuo et al., 2014).

The C terminal of TDP-43 is highly disordered, binds other RNA binding proteins (RBPs) and drives aggregation (Johnson et al., 2009; Fuentealba et al., 2010). Recent evidence shows that TDP-43 binds to Fragile X mental retardation protein (FMRP), another RBP, to co-repress translation (Majumder et al., 2016). The intrinsically disordered region (IDR) of TDP-43 could drive LLPS and eventual aggregation. LLPS is the process responsible for the formation of dynamic, round, protein rich complexes that can undergo fusion similar to *P* granules or nucleoli. Working with six different RBPs, Lin et al. recently demonstrated that IDRs can undergo phase separation on their own or in concert with RNA-binding domains. IDRs also regulate the recruitment of other RBPs to phase separated droplets (Lin et al., 2015). In addition, the intrinsically disordered, aggregation prone C terminus contains a prion like region, deletion of which can prevent aggregation and mis-folding events (Fuentealba et al., 2010; King et al., 2012). Interestingly, recent structural analyses have shown that the C terminus domain can also bind nucleic acids (i.e., ssDNA) as well as membranes, suggesting that it can provide a complex energetic landscape influencing the conformation of this critical region of TDP-43 (Lim et al., 2016). In addition, 4-aminoquinolines that were shown to disrupt nucleic acids–TDP-43 interactions *in vitro*, also seem to modulate the association of TDP-43 with ubiquillin 2, which occurs via its C terminus domain (Cassel et al., 2012; Cassel and Reitz, 2013). These findings suggest that the unstructured C terminal domain may participate in and/or modulate the association of TDP-43 with nucleic acids.

### TDP-43 Localization

In one of the earliest studies of TDP-43, microscopy and fractionation approaches showed that although endogenous TDP-43 can shuttle between the nucleus and the cytosol it is primarily nuclear, with a small fraction of the protein localizing to the cytoplasm (Ayala et al., 2008). Mutations that disrupt RNA binding by the RRM domains lead to restriction of the majority of TDP-43 to the nucleus suggesting that its association with RNA dictates its export into the cytoplasm (Ayala et al., 2008; Elden et al., 2010). Overexpression leads to increased TDP-43 levels in the cytoplasm and is sufficient to cause toxicity in yeast (Johnson et al., 2008). Consistent with this observation, the presence of TDP-43 in the cytoplasm is sufficient to cause ALS like phenotypes in mice (Walker et al., 2015). Evidence exists to support both a nuclear loss of function and a cytoplasmic gain of function in disease (reviewed in Vanden Broeck et al., 2014). Further substantiating the presence of both loss and gain of function mechanisms are findings that expressing human TDP-43 without a nuclear localization signal (ΔNLS) in mice causes, among other ALS phenotypes, a nuclear loss of endogenous mouse TDP-43, and doxycycline induced removal of ΔNLS TDP-43 restores neuromuscular phenotypes to normal, respectively (Walker et al., 2015).

Patients with frontotemporal lobar degeneration (FTLD-U) or ALS exhibit ubiquitinated, tau-negative inclusions localized to the cytoplasm and containing TDP-43 that are distinct from amyloid deposits seen in other neurodegenerative disorders (Neumann et al., 2006). Immunohistochemistry and Western blot analyses using patient tissues showed that pathological TDP-43 inclusions in the cytoplasm are also abnormally hyperphosphorylated, and TDP-43 is cleaved to generate C terminal fragments of ~25 kDa (Kabashi et al., 2008). Another cytoplasmic fragment associated with pathology is approximately 35 kDa, represents an N-terminally truncated splice variant of was found to be upregulated in ALS (Nishimoto et al., 2010; Xiao et al., 2015). The 35 kDa fragment has been shown to exhibit reduced solubility and cytoplasmic aggregation and is derived from an alternative translation initiation site, specifically ATG^Met85^ (Xiao et al., 2015). Interestingly, C-terminal fragments can act as seeds to recruit full length TDP-43 to cytoplasmic aggregates and cause TDP-43 depletion from the nucleus, providing a possible scenario for prion like propagation of disease (Nonaka et al., 2013).

### The Roles of TDP-43 in RNA Processing: Splicing, mRNA Transport, Stability, and Translation

Since the first demonstration of its role in the splicing of the CFTR gene (Buratti et al., 2001) many more splicing targets of TDP-43 were identified using knock-down accompanied by RNA seq (Polymenidou et al., 2011) or UV-crosslinking followed by immunoprecipitation (CLIP) (Tollervey et al., 2011). These experiments showed that TDP-43 can bind both introns and exons. Interestingly, intronic targets were enriched for genes regulating neurotransmitter processes as well as synaptic formation and function. In addition to UG tandem repeats or long clusters of UG rich motifs, TDP-43 also binds non-coding RNAs, introns and 3′ UTRs of mRNAs. Further substantiating a role for TDP-43 in several RNA processes are elegant Bru-seq experiments showing a role for TDP-43 in mRNA stability and mass spectrometry studies of protein complexes showing that proteins that copurify with TDP-43 are functionally associated with RNA splicing and transport (Freibaum et al., 2010; Sephton et al., 2011; Tank et al., 2018).

The functional significance of TDP-43's role in splicing was elegantly shown in *Drosophila* loss of function mutants using RNA seq (Hazelett et al., 2012). Gene ontology analyses of the genes that are differentially expressed in the *Drosophila* loss of function mutants for TDP-43 (TBPH^−/−^) highlight alterations in synaptic transmission, neurotransmitter release and endocytosis with several ligand- or neurotransmitter-gated ion-channels and neuropeptide receptors being misexpressed. Furthermore, loss of TBPH leads to altered splicing of transcripts including the voltage-gated calcium channel, *cacophony*. A subsequent study showed that null mutants for TBPH cause locomotor deficits and reduced amounts of cacophony protein at the neuromuscular junctions in Drosophila (Chang et al., 2014). Notably, expression of cacophony, specifically in the motor neurons rescued the locomotor deficits in Drosophila TBPH mutants, highlighting the functional significance of splicing alterations caused by nuclear loss of TDP-43 (Chang et al., 2014).

Further substantiating the relationship between splicing and TDP-43 toxicity is the finding that loss of function for *Dbr1*, an RNA lariat debranching enzyme, or inhibition of its enzymatic activity suppresses TDP-43 dependent phenotypes in yeast and mammalian neurons (Armakola et al., 2012). In the absence of *Dbr1* function, intronic lariats accumulate in the cytoplasm possibly acting to sequester TDP-43 and prevent it from interacting with essential RNAs and RNA-binding proteins that in turn causes toxicity. In addition, loss of function studies show that the role of TDP-43 in splicing also includes maintaining intron integrity by suppressing cryptic exons (Ling J. P. et al., 2015).

### mRNA Localization and Translation

Although the majority of TDP-43 is normally localized to the nucleus, a small amount is present in the cytoplasm (Ayala et al., 2008). Motivated by reports that TDP-43 mislocalization from the nucleus to the cytoplasm constitutes an early event in ALS pathogenesis (Giordana et al., 2010), several groups generated disease models based on overexpression of TDP-43 in yeast, worms, flies, fish, and mice (Johnson et al., 2008; Kabashi et al., 2009; Wegorzewska et al., 2009; Liachko et al., 2010; Li et al., 2010). Indeed, overexpression of full length TDP-43, disease associated variants or a TDP-43 mutant lacking the nuclear localization sequence led to mostly cytoplasmic localization of TDP-43 and formation of puncta, which mimicked pathological inclusions in flies (Lu et al., 2009; Li et al., 2010; Estes et al., 2011, 2013), worms (Liachko et al., 2010) or rat primary cortical neurons (Barmada et al., 2010). Overexpressed RRM mutants on the other hand did not localize to the cytoplasm and did not cause toxicity (Elden et al., 2010; Voigt et al., 2010; Ihara et al., 2013; Coyne et al., 2015). Notably, overexpression is not the only way to generate ALS like phenotypes. In keeping with the view that pathology is likely a combination of loss of nuclear function and gain of cytoplasmic function, null mutants have been shown to have locomotor defects, reduced lifespan, and abnormal neuromuscular junctions in Drosophila (Feiguin et al., 2009). Furthermore, conditional knock-out of TARDBP (Wu et al., 2012) or expression of disease associated mutant TDP-43 at close to endogenous levels [which caused neither loss of nuclear localization of cytoplasmic aggregation (Arnold et al., 2013; Gordon et al., 2019)] resulted in ALS-like phenotypes in mice. In further support of co-existing nuclear loss and cytoplasmic gain of function mechanisms, mouse embryonic fibroblasts derived from mice expressing an RRM2 mutant form of TDP-43 exhibited splicing changes similar to TDP-43 knockdown (i.e., inclusion of cryptic exons) while the expression of C terminus mutations caused gain of function mis-splicing (i.e., skiptic exons; Fratta et al., 2018). In contrast, loss of TDP-1 in worms causes an extension of lifespan (Zhang et al., 2012). Taken together, these studies showed that TDP-43 toxicity can be caused by several mechanisms including cytoplasmic mislocalization, and is dose and RNA dependent.

How might cytoplasmic TDP-43 regulate RNA processing and contribute to toxicity? According to the ribostasis hypothesis (Ramaswami et al., 2013), cytoplasmically mislocalized TDP-43 associates with RNA-containing stress granules (SGs) or other types of RNA granules (e.g., transport granules). Then, perhaps due to stress caused by aging, the presence of mutations or environmental insults, cytoplasmic TDP-43 complexes could become insoluble and trap RNA and/or proteins causing disturbances in both ribostasis and proteostasis, followed by motor neuron dysfunction and death. Supporting this hypothesis, a number of studies have shown that when overexpressed, TDP-43 alters the transport, localization, and translation of specific mRNAs in both axons and dendrites (Wang et al., 2008; Fallini et al., 2012; Majumder et al., 2012, 2016; Alami et al., 2014; Coyne et al., 2014, 2017a; Liu-Yesucevitz et al., 2014). For example, mislocalization, sequestration, and reduced translation of mRNAs encoding the microtubule stabilizing protein Futsch/MAP1B and/or hsc70-4/HSPA8 have been shown to mediate TDP-43 dependent toxicity in Drosophila (Coyne et al., 2014, 2017a). Interestingly, patient derived motor neurons and spinal cords showed similar changes in hsc70-4/HSPA8 and Futsch/MAP1B protein levels, respectively, suggesting that altered mRNA transport, localization, and translation may contribute to disease pathogenesis.

## Cytoplasmic TDP-43 Assemblies

A significant body of work since the discovery of TDP-43 highlights the presence of a wide array of protein/RNA complexes containing TDP-43 (Colombrita et al., 2009; Freibaum et al., 2010; Liu-Yesucevitz et al., 2010; Nishimoto et al., 2010; Dewey et al., 2011; McDonald et al., 2011; Sephton et al., 2011). While a definitive understanding of the relationship between the different types of TDP-43 containing complexes and their involvement in disease is currently lacking, evidence exists to support the presence of multiple types of TDP-43 protein assemblies, some of which are soluble, while others are insoluble (aggregate-like) and some that contain RNA while others do not. As discussed below, although a substantial amount of work supports the ribostasis hypothesis, a series of recent studies provoke a reevaluation of the precise mechanisms by which TDP-43 contributes to disease and the significance of RNA association with TDP-43 (McGurk et al., 2018; Zhang et al., 2018, 2019; Fang et al., 2019; Gasset-Rosa et al., 2019; Mann et al., 2019).

### RNA Stress Granules

**Figure 1:**
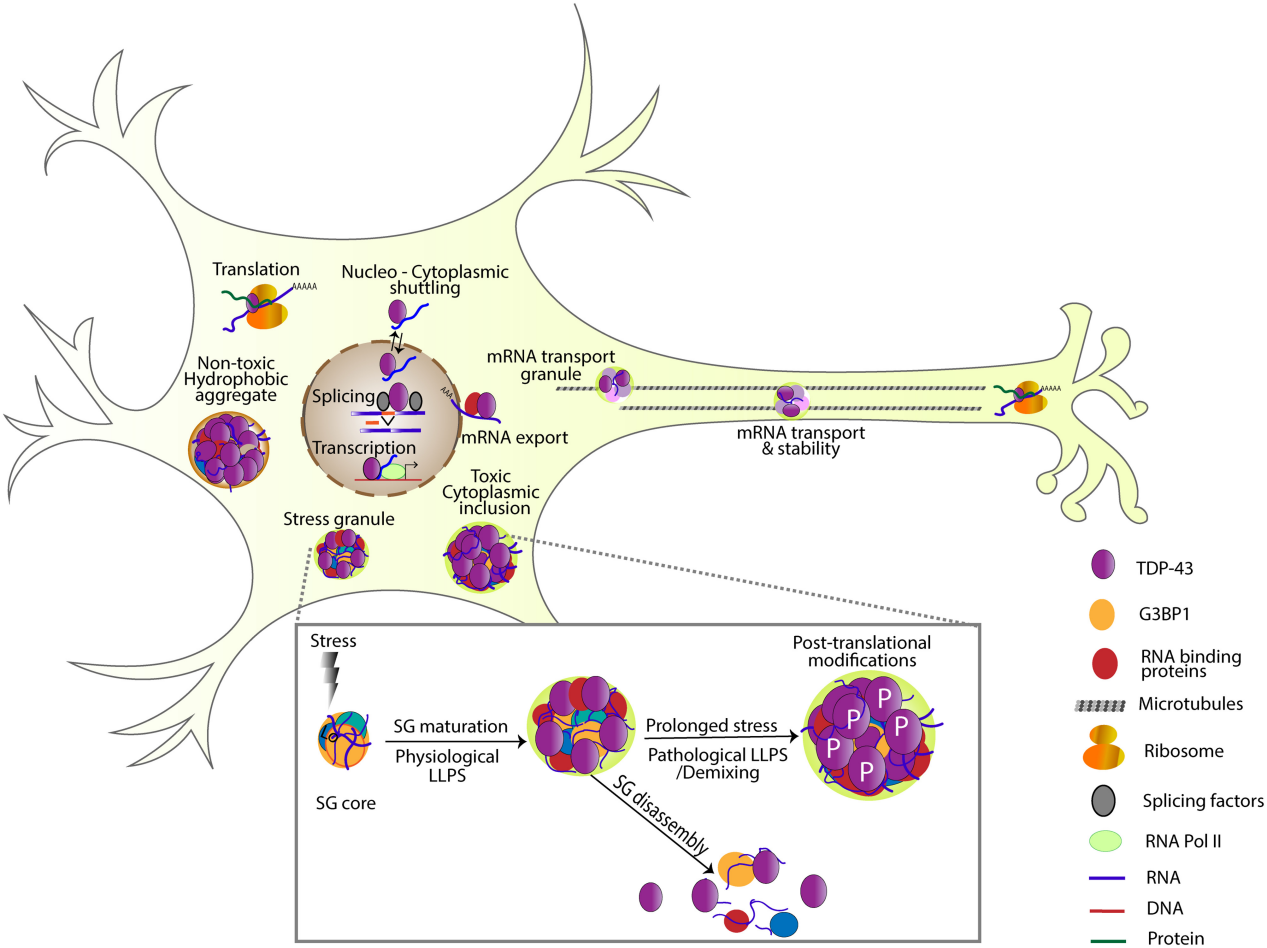
TDP-43 is involved in several steps of RNA processing including transcription, splicing, RNA stability, mRNA transport, localization, translation, and SG formation. Under transient stress, protein-protein and RNA interactions are nucleated by G3BP1, forming a dense SG core. TDP-43 and other RNA binding proteins are recruited to these cores and undergo liquid-liquid phase separation forming a dynamic shell structure (physiological LLPS). Under transient stress, SGs disassemble, and return these RNA binding proteins to their normal function. Under prolonged stress, TDP-43 undergoes post-translational modifications like phosphorylation, and becomes insoluble (pathological LLPS). Note non-toxic hydrophobic aggregates and toxic cytoplasmic inclusions as examples of different TDP-43 complexes that have been detected in cells (Bolognesi et al., 2019).

Once in the cytoplasm, TDP-43 can associate with several types of RNA granules and/or protein assemblies including stress granules, transport Ribonucleoprotein particles (RNPs) as well as insoluble protein complexes. SGs are non-membrane bound organelles that assemble when translation initiation is inhibited or during stress (e.g., heat shock, osmotic pressure, oxidative stress; Kedersha and Anderson, 2007; Buchan and Parker, 2009). SGs provide a mechanism for adaptive response to stress (Riback et al., 2017) and have recently been shown to preferentially sequester long, poorly translated RNAs as well as a diverse set of proteins including nuclear pore components and RBPs in a cell and stress type dependent manner (Khong et al., 2017; Markmiller et al., 2018; Namkoong et al., 2018; Zhang et al., 2018). Observations of SG formation have led to a multistep model involving the microtubule cytoskeleton (Fujimura et al., 2009), however more recently SG formation has been refined a two-phase assembly model (Wheeler et al., 2016). First, an established network of protein-protein and RNA interactions, nucleated by G3BP1, rapidly assemble to form a dense core structure where RNA-protein exchange is modulated by ATPases. RBPs, many of which contain aggregate-prone intrinsically disordered regions, are recruited to these cores through RNA-interactions and this increase in their local concentration triggers liquid-liquid phase separation (LLPS) leading to the formation of a more dynamic shell structure (Jain et al., 2016; Wheeler et al., 2016; Markmiller et al., 2018; Fang et al., 2019; Figure 1). Structure function studies have shown that both RNA binding and the C terminus domain are required for TDP-43 association with SGs (Colombrita et al., 2009; McDonald et al., 2011). Loss of TDP-43 does not abolish SG assembly during oxidative stress. Instead, it prevents the formation of larger SGs implicated in the protection of polyadenylated RNA during oxidative stress, by down-regulating G3BP1 (Aulas et al., 2012, 2015). Disease associated TDP-43 variants (A315T and Q343R) but not NLS domain mutants (K82/84A) also modulate SG dynamics in primary hippocampal neurons or iPSCs derived motor neurons by increasing the average size, decreasing the distribution density, and reducing the mobility of SGs compared to wild-type controls (Liu-Yesucevitz et al., 2014). Consistent with these findings, mutant TDP-43 mislocalizes to the cytoplasm and causes a significant reduction in SG size in embryonic stem cell-derived primary motor neurons (Gordon et al., 2019). Interestingly, while TDP-43 regulates the dynamics of SGs in neurons and astrocytes under different stressors, hyperosmotic stress induced by 1M D-sorbitol impairs SG assembly in astrocytes but not neurons lacking TDP-43 (Colombrita et al., 2009; Liu-Yesucevitz et al., 2010; Dewey et al., 2011; McDonald et al., 2011; Khalfallah et al., 2018). Also, of note is the fact that SGs in astrocyte are found closer to the cell periphery similar to the localization of TDP-43 puncta in Drosophila glia (Estes et al., 2013; Khalfallah et al., 2018).

In turn, the impact of SGs on TDP-43 and disease pathomechanisms is less clear. It was recently shown that increased cytoplasmic levels of TDP-43 are sufficient to induce long-lived liquid-liquid phase separation (LLPS) and toxic inclusions that disrupt nuclear transport (Gasset-Rosa et al., 2019). This points to SGs having no role in the formation of toxic TDP-43 inclusions although a small fraction of SGs induced by arsenite was found to contain TDP-43. More in line with the widely accepted view of SGs as having a protective role, the association of TDP-43 with SGs was reported to be neuroprotective (McGurk et al., 2018). Mechanistically, the association of TDP-43 with SGs, through its Poly(ADP-ribose) polymerase (PAR) binding domain, initially prevents phosphorylation, which has been associated with TDP-43 toxicity and disease. Further substantiating the protective role of SGs are findings that RNA binding prevents LLPS of opto-TDP-43 and recruits TDP-43 to SGs, which increases its solubility during transient stress compared to TDP-43 not found in RNA-containing assemblies (Mann et al., 2019). A common conclusion among these recent studies is that short-term association to SGs may be neuroprotective for TDP-43, presumably because of their high concentrations of RNA available to bind, while the repeated, long-term formation of SGs has negative consequences to neuronal viability (McGurk et al., 2018; Mann et al., 2019).

Since the report of core SG components colocalizing with TDP-43 in pathological inclusions (Liu-Yesucevitz et al., 2010), the strongest evidence that SGs represent precursors of TDP-43 containing pathological aggregates comes from recent studies involving Opto-SGs (Zhang et al., 2019). Studies using Opto-G3BP1 that assemble SGs upon blue-light treatment showed that over time, following continuous or repeated transient stress conditions, OptoSGs accumulate increasing levels of TDP-43, A11, ubiquitin, and disease-associated phosphoTDP-43. These findings support a model whereby repeated close packing of these proteins seeds pathological amyloids especially for proteins like TDP-43, which can adopt highly stable structures. In addition, the chronic induction of SGs in iPSC-derived neurons leads to altered SG dynamics, loss of cell viability and the evolution of disease-like TDP-43 cytoplasmic inclusions (Zhang et al., 2019). Interestingly, a screen for small-molecule compounds that alter SG dynamics led to findings that disrupting the RNA interactions that recruit RBPs into SG shells can prevent the association and thereafter aggregation of mutant ALS-related proteins such as TDP-43 during chronic stress (Fang et al., 2019). This further substantiates findings that TDP-43 associates with SGs, which at least in some experimental settings (e.g., chronic stress conditions) can “evolve” into TDP-43 containing insoluble complexes, consistent with the ribostasis hypothesis. However, the association of TDP-43 with SGs seems to be both toxic and protective depending on the experimental model and approach (transient vs. chronic, and type of stress), requiring further study. Ultimately, disease is likely to result from the simultaneous disruption in multiple LLPS forming molecular complexes as suggested by findings that SGs sequester nuclear pore proteins, which in turn alter the dynamics of the nuclear pore (Zhang et al., 2018), possibly propagating alterations throughout the cellular network (Zhang et al., 2019).

### RNA Transport Granules

TDP-43 has been implicated in axonal and dendritic transport (Wang et al., 2008; Alami et al., 2014). More recently, high resolution imaging approaches provided evidence for liquid like properties of TDP-43 containing RNP granules *in situ*. Using rodent primary cortical neurons, they demonstrated demixing and identified distinct biophysical properties of RNP granules depending on axonal location and TDP-43 variant (wild-type vs. disease associated mutant). TDP-43 RNA granules were more spherical and more dynamic in distal end than in proximal axons. Additionally, RNP transport granules formed in ALS associated TDP-43 mutant models were more viscous and showed disrupted dynamics. Notably, when RRM domains were mutated, the neurons were devoid of TDP-43 puncta suggesting that RNA binding is required for granule formation in axons (Gopal et al., 2017). This is consistent with several studies showing that impairing the ability of the RRM domains to bind RNA results in reduced or no toxicity (Elden et al., 2010; Voigt et al., 2010; Ihara et al., 2013; Flores et al., 2019).

### Myo-granules

Recently, TDP-43 was shown to be essential for normal skeletal muscle formation and regeneration. TDP-43 localizes to sites of sarcomere formation, and forms cytoplasmic aggregates that show amyloid like features (Vogler et al., 2018). Interestingly, these aggregates called myo-granules, are cleared as myofibrils mature. While myo-granules appear to be physiological aggregates, pathological TDP-43 aggregates can be found in skeletal muscle diseases such as Inclusion Body Myositis (IBM) (Salajegheh et al., 2009), which are similar to aggregates found in neurons. The presence of these otherwise functional aggregates provides an opportunity for seeding and formation of muscle pathological aggregates that have been observed in disease (Vogler et al., 2018).

## A Role for RNA in TDP-43 Mediated Toxicity

### RNA as a Toxic Factor

Several papers have shown that RNA binding is required for TDP-43 mediated toxicity *in vivo* (Elden et al., 2010; Voigt et al., 2010; Ihara et al., 2013). Consistent with a role for RNA in TDP-43 toxicity, a recent study found that RNA binding to TDP-43 is positively correlated with toxicity and reduced TDP-43 turnover and/or increased TDP-43 stabilization (Flores et al., 2019). Using mouse primary cortical neurons, they determined that RNA bound to RRMs is necessary, but not sufficient for toxicity. Overexpression of either wild-type TDP-43 or TDP-43 containing RRMs from other proteins resulted in reduced TDP-43 turnover and increased toxicity relative to the overexpression of a variety of TDP-43 mutants which are unable to bind RNA. Additionally, overexpression of RNA binding deficient TDP-43 did not induce the transcriptional changes in mitochondrial and ribosomal transcripts that were observed as a result of wild-type TDP-43 overexpression. Mechanistically, proteasome inhibition significantly mitigated the correlation between RNA binding and TDP-43 turnover and attachment of a destabilizing residue chain to the C terminal domain of TDP-43 increased the rate of TDP-43 turnover. These results suggest that RNA binding stabilizes TDP-43 and reduces the rate of proteasome dependent degradation, which in turn enhances toxicity (Flores et al., 2019).

Recent studies have shown that TDP-43 RRMs can contribute to TDP-43 aggregation, with RRM1 capable of aberrant self-assembly (Zacco et al., 2018) and aggregation prone conformations of RRM2 more abundant in the context of oxidative stress or TDP-43 over expression (Tavella et al., 2018). These results suggest that the TDP-43 RRMs can alter TDP-43 self-assembly dynamics independent of RNA. Furthermore, a recent study using a combination of biophysical approaches showed that both sequence and length of RNA can influence TDP-43 aggregation. Specifically, long UG repeats may help stabilize TDP-43 structure while excess non-specific RNA may promote aggregation (Zacco et al., 2019). Taken together these studies underscore the importance of a thorough characterization of the different TDP-43 conformations in the context of its RNA and protein partners.

Supporting a role for RNA as a factor inducing toxicity is the recent identification of small molecules that can bind TDP-43 and displace G quadruplex like structures but not canonical UG repeats. Notably, one of these structures identified using *in silico* design also mitigates TDP-43 dependent toxicity *in vivo*, in a *Drosophila* model of ALS (Francois-Moutal et al., 2019). Although it remains to be determined whether this small molecule works *in vivo* by actually binding TDP-43 and displacing mRNA targets, the findings provide proof of principle that targeting the RRM domain of TDP-43 may provide strategies for mitigating toxicity at least in the context of TDP-43 overexpression.

Another piece of evidence that displacing RNA interactions can be protective comes from a recent small molecule screen aimed at modifying SG dynamics. Testing >9,000 small molecules for their ability to modify SG assembly and/or disassembly led to the identification of a set of planar molecules that interact with RNA in SGs and also dislodge RNA binding proteins such as TDP-43 form their association with SGs (Fang et al., 2019). These same molecules appear to improve survival in TDP-43 overexpressing mouse motor neurons and in patient iPSC derived motor neurons. Whether these structures improve survival due to binding RNA and displacing TDP-43 from SGs seeding aggregates or through an alternative mechanism, remains unknown at this time.

### RNA as a Protective Factor

*In vitro* binding data shows that adding RNA containing UG repeats inhibits TDP-43 oligomerization and aggregation, suggesting that TDP-43 solubility depends on the availability of mRNA containing this motif (French et al., 2019). Supporting these findings is a recent report that RNA inhibits LLPS for prion domain containing RNA binding proteins, thus preventing maturation into insoluble complexes and pathological aggregates (Maharana et al., 2018). This explains why in the nucleus, where RNA is abundant, RNA binding proteins such as TDP-43 are diffuse while in the cytoplasm where the RNA/protein ratio is lower, TDP-43 forms aggregate-like structures. Indeed, RNAse treatment induces FUS inclusions in the nucleus (Maharana et al., 2018). Consistent with these reports, it was recently found that RNA binding antagonizes TDP-43 toxicity and cytoplasmic puncta formation (Mann et al., 2019). Using elegant, optogenetically induced opto-TDP granules in human embryonic kidney (HEK) cells and human cultured neurons, the ability of TDP-43 to bind RNA was negatively correlated with toxicity and TDP-43 insoluble complex formation. TDP-43 containing complexes containing mRNA and stress granule markers (e.g., G3BP1 and EIF4G) were significantly more dynamic than their mRNA lacking counterparts, suggesting that in the absence of RNA binding, TDP-43 forms insoluble mRNA-less complexes, which contribute to toxicity. Indeed, utilizing FISH (fluorescent *in situ* hybridization), mRNA was determined to be absent from many TDP-43 pathological complexes. Lastly, the correlation persisted when TDP-43 RRMs were replaced with the RRM of another RNA binding protein, FUS, suggesting that general RNA binding is sufficient for mitigating toxicity.

In support of these recent findings, transient stress was shown to induce long lasting TDP-43 phase separation in the cytoplasm and was independent of mRNA (Gasset-Rosa et al., 2019). The authors demonstrated that TDP-43 expressed at endogenous levels phase separates into liquid like rounded particles under physiological conditions. In response to transient stress, these granules redistribute to the cytoplasm and demix into liquid like soluble granules independent of SGs. Under prolonged stress, the liquid like granules form solid insoluble aggregates devoid of any detectable mRNA while at the same time showing disease associated phosphorylation of TDP-43. Once formed, cytoplasmic aggregates cause eventual depletion of nuclear TDP-43 and induce cell death.

The conflicting studies regarding the relationship between TDP-43 toxicity and RNA binding suggest that there are yet-to-be-understood confounding factors which modify the interaction between TDP-43 toxicity and RNA such as post-translational modifications. Supporting this scenario is a recent report showing that PARylation of TDP-43 regulates its recruitment to SGs via PAR binding motifs (PBMs) located within the NLS (McGurk et al., 2018). Indeed, PBM disruption leads to disease associated phosphorylation of TDP-43. Further substantiating this scenario are findings that pharmacological inhibition of tankyrase, a PAR polymerase reduces TDP-43 cytoplasmic foci and mitigates neurodegeneration in flies (McGurk et al., 2018).

## Discussion

Although several recent publications demonstrate that phosphoTDP-43 containing pathological aggregates can form and persist independently of SGs, the evolution of toxic TDP-43 complexes from SGs remains a strong possibility supported by some of the same studies and many others. Examining the evidence as a whole, it appears that at least transiently, TDP-43 associates with RNA containing complexes (e.g., SGs, transport granules), however, under persistent stress these assemblies may undergo LLPS and promote the formation of insoluble TDP-43 complexes some of which contain RNAs and some which do not. It is likely that distinct RNA types (e.g., UG repeat containing, G quadruplex forming, etc.) have different effects on TDP-43 solubility in cells. We speculate that the type and quantity of RNA available to interact with TDP-43 together with protein-protein interactions shape the type of assembly that TDP-43 associates with, and provide an explanation for the observed heterogeneity. Just reported, a comprehensive mutagenesis screen in yeast identified a 31 amino acid “hot-spot” (312−342) where mutations can cause different degrees of toxicity (Bolognesi et al., 2019). Interestingly, mutations that increase hydrophobicity and aggregation were found to be less toxic than those that promote LLPS. Ultimately, elucidating the connection between RNA, protein association and TDP-43 toxicity are needed to understand which assemblies are protective vs. toxic and may guide rational drug design. Regardless of whether RNA is present or not, restoring the solubility of TDP-43, associated RNAs and protein partners to ensure correct levels of gene expression remains a viable solution for mitigating TDP-43 toxicity (Figure 1).

## Author Contributions

SL, EL, RE, and DZ wrote the manuscript. SL made the figure.

### Conflict of Interest

The authors declare that the research was conducted in the absence of any commercial or financial relationships that could be construed as a potential conflict of interest.
